# LiDAR-Stabilised GNSS-IMU Platform Pose Tracking

**DOI:** 10.3390/s22062248

**Published:** 2022-03-14

**Authors:** Timothy D’Adamo, Tyson Phillips, Peter McAree

**Affiliations:** School of Mechanical and Mining Engineering, The University of Queensland, Brisbane, QLD 4072, Australia; t.phillips1@uq.edu.au (T.P.); p.mcaree@uq.edu.au (P.M.)

**Keywords:** GNSS, IMU, LiDAR, perception, pose estimation, navigation system, terrain mapping

## Abstract

The requirement to estimate the six degree-of-freedom pose of a moving platform frequently arises in automation applications. It is common to estimate platform pose by the fusion of global navigation satellite systems (GNSS) measurements and translational acceleration and rotational rate measurements from an inertial measurement unit (IMU). This paper considers a specific situation where two GNSS receivers and one IMU are used and gives the full formulation of a Kalman filter-based estimator to do this. A limitation in using this sensor set is the difficulty of obtaining accurate estimates of the degree of freedom corresponding to rotation about the line passing through the two GNSS receiver antenna centres. The GNSS-aided IMU formulation is extended to incorporate LiDAR measurements in both known and unknown environments to stabilise this degree of freedom. The performance of the pose estimator is established by comparing expected LiDAR range measurements with actual range measurements. Distributions of the terrain point-to-model error are shown to improve from 0.27 m mean error to 0.06 m when the GNSS-aided IMU estimator is augmented with LiDAR measurements. This precision is marginally degraded to 0.14 m when the pose estimator is operated in an *a prior* unknown environment.

## 1. Introduction

An inertial measurement unit (IMU) aided by global navigation satellite system (GNSS) information is often used to track the six degree of freedom (DOF) pose (here, pose refers to the six degree of freedom position (*x*, *y*, *z*) and orientation (θ, ϕ, ψ) of the platform) of a moving platform. Solutions come in many forms but are broadly categorised as being tightly or loosely coupled depending on the level of sensor integration. The rationale for using the two sensor types is that GNSS receivers provide low-frequency information while the IMU provides information about higher frequency motion, noting that when stationary, the IMU measures gravity which can be considered as zero-frequency (DC) information. In a typical implementation, measurements from the two sensors are combined using optimal estimation methods, vis-à-vis Kalman filtering, to maintain an estimate of the pose of the platform frame *P* as it moves relative to a global frame *G*.

Where three GNSS receivers with non-collinear antenna are used, the low-frequency pose of the platform can be fully determined to the precision and bandwidth of the receivers. Each GNSS receiver provides positional knowledge of its antenna centres to a precision of approximately 0.05 m at a bandwidth from 0 Hz to 2–5 Hz (with measurement updates typically provided at 10 Hz). Higher frequency motion is then determined from information provided by the IMU, which will typically provide acceleration and angular rate information from 0 Hz to 50 Hz, at update rates of 50 Hz to 100 Hz. Such an arrangement provides a nice division of labour, with the Kalman filter-based pose estimator serving to combine the disparate frequency information provided by the two sensor types.

However, where two GNSS receivers are used, only five of the six degrees of freedom of the platform can be established using the information provided by the GNSS receivers. The platform’s rotational orientation about the line drawn through the antenna centres is not defined. In theory, gravity can be used to resolve this, provided the placement of the IMU frame does not result in the vector emanating from the sensor origin and directed by gravity, intersecting the antenna centre line. In practice, however, for a similarly structured estimator, start-up bias and the conflation of gravity measurement with accelerations due to platform motion limits the accuracy of pose estimates.

This paper develops and evaluates a method for using three-dimensional LiDAR measurements to stabilise pose estimates for platforms having two GNSS receivers. We consider two situations: (i) where the environment around the platform is *a priori* known, and (ii) where it is not. LiDAR sensors are often present on robotic platforms as components of a perception system. Their use for stabilising pose estimates becomes relevant where three GNSS receivers are impractical or cost-prohibitive.

Pose estimation is clearly ground for fertile research and has been for some time. Google Scholar identifies no fewer than 4650 publications that contain all of the terms “GNSS”, “IMU”, and “loosely coupled”. A total of 851 of these were published after 2020; the first was published in 1968 [1]. If the term “pose” is included, 1050 papers are identified; 279 of these were published after 2020, while the earliest is [2]. Adding the term “LiDAR” reduces the set to 452, of which 167 have been published since 2020, with the earliest again dating from 1981 [3]. These, and the many other papers that use alternative but equivalent terminology, span a variety of approaches and applications, from terrestrial [4,5,6], to aeronautical [7,8,9,10,11], space [12,13,14,15]. In some instances, the search terms appear in reference to the technology being used for a specific application, e.g., precision agriculture [16,17,18,19] and construction [20,21]. In others, reference is made to implementation and algorithms. These are described at various levels of detail, but often with ambiguity.

Methods for stabilising pose estimates with LiDAR measurements exist and generally fall into two categories. The first are *feature-based* methods which seek to identify features, sometimes known as landmarks, in LiDAR point clouds and maintain an estimate of their location. Features can be lines or planes [22,23,24], curves [25,26], or corners [27]. Feature-based methods can also match sequential LiDAR scans, solving for the incremental change in pose between scans [28,29,30,31,32].

An alternative approach uses *model-based* methods. These generally compare measurements from a ranging sensor with a model of the environment, searching for a pose solution that provides an optimal solution.

Ref. [33] propose an airborne solution for aiding a navigation system through the use of a large-scale terrain model and LiDAR sensors. Similarly, Ref. [34] augment pose estimates by fitting LiDAR measurements against a known two-dimensional environment model. Refs. [35,36] show how pose estimates of known objects can be obtained from only LiDAR scans by finding the pose that is most likely among the set of all possible poses.

Other work has focused on the augmentation of pose when GNSS measurements are degraded or unavailable, such as loss of real-time kinematic (RTK) corrections. Ref. [37] develop a solution that uses a known geometric model of the world and that augments GNSS measurements with a pseudo-measurement of the expected height of the receiver. Ref. [38] use a conceptually similar approach, but apply it to aerial vehicles.

Work on *model-based* methods has also focused on building an environment model simultaneously with localisation within this model. One solution, presented by [39], develops an algorithm for a LiDAR- and GNSS-aided IMU navigation system for use in an airborne platform that only maps the terrain when GNSS and IMU measurements are available and reliable. When the integrity of the GNSS-aided IMU navigation system is compromised, LiDAR observations are fitted to the generated terrain model through an ICP-based method. The authors find that the solution is robust and improves the accuracy of the pose when the GNSS system is in a degraded state.

Ref. [40] present a method that augments a GNSS-aided IMU and odometry navigation system with a scanning LiDAR sensor. The authors estimate the environment using the LiDAR scanner as they move through an urban scene, and remove dynamic obstacles by compressing this model to a ground plane, coloured based on infrared reflectivity from LiDAR return intensities. They identify that their novel navigation system is up to an order of magnitude more accurate than a GNSS-aided IMU and odometry navigation system.

Our rationale for writing this paper is twofold. First, we believe the approach we propose for the integration of LiDAR measurement into a GNSS-aided IMU solution is novel. Second, we have repeatedly sought clear descriptions of the algorithms others have used, only to find missing details or gaps that have frustrated our implementation efforts. Here, we attempt to give a full and complete description of a loosely coupled GNSS-aided IMU navigation system in the expectation that it will be useful to others.

## 2. Experimental Platform

**Figure 1:**
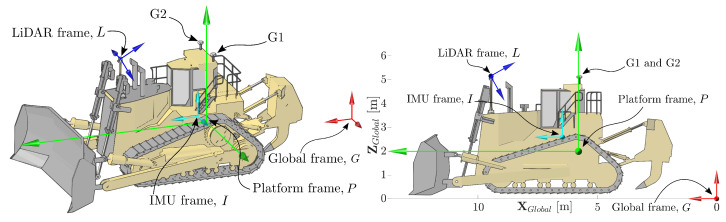
The Caterpillar D11T bulldozer considered in this paper, showing the sensors installed on the platform. The platform is equipped with two GNSS receivers, one IMU, and a scanning LiDAR sensor that faces forward and scans around a horizontal axis. The frames referred to in this paper are labelled as the LiDAR frame, *L*, the IMU frame, *I*, the platform frame, *P*, and the global frame, *G*.

Figure 1 shows the experimental platform considered in this paper. The machine is called a track-type tractor or bulldozer and has applications in agriculture, construction, and mining. In all these domains, tracking spatial pose is needed for technology applications that include production reporting, guidance, and automation.

Figure 1 shows the coordinate frames in which variables relevant to the development of the pose estimation system are represented. The global frame is denoted as *G*. Frame *P* describes the pose of the tractor in Frame *G* by a transformation $T_{P}\left(\;\cdot \;,\;\cdot \;\right)$. Sensors are described relative to Frame *P*: Frame *I* describes the position and orientation of the IMU; Frame *L* describes the position and orientation of a LiDAR; and $G_{1}$ and $G_{2}$ are the centres of the antennas of two GNSS receivers mounted on the tractor cabin.

The pose of the tractor is defined by the state vector,

(1) $$x={\left[\begin{array}{ccccccc} p_{P} & {\dot{p}}_{P} & {\ddot{p}}_{P} & \Phi_{P} & \omega_{P} & b_{a} & b_{r} \end{array}\right]}^{T}.$$

The 21 state parameters of the state vector are intended to accommodate a constant acceleration model for translation and a constant rate model for attitude. Here,

$p_{P}={\left[x,y,z\right]}^{T}$ is the coordinate of the origin of *P* in *G*;
${\dot{p}}_{P}={\left[\dot{x},\dot{y},\dot{z}\right]}^{T}$ is the velocity of point $p_{P}$ in *G*;
${\ddot{p}}_{P}={\left[\ddot{x},\ddot{y},\ddot{z}\right]}^{T}$ is the acceleration of point $p_{P}$ in *G*;
$\Phi_{P}={\left[\theta ,\phi ,\psi \right]}^{T}$ describes the attitude of Frame *P* in *G* as roll, pitch and yaw;
$\omega_{P}={\left[\omega_{x},\omega_{y},\omega_{z}\right]}^{T}$ describes the rotational velocity of Frame *P* in *G*;
$b_{a}={\left[b_{\ddot{x}},b_{\ddot{y}},b_{\ddot{z}}\right]}^{T}$ describes the acceleration bias of the IMU in Frame *I*;
$b_{r}={\left[b_{\omega_{x}},b_{\omega_{y}},b_{\omega_{z}}\right]}^{T}$ is the rotational rate bias of the IMU in Frame *I*.

The transformation matrix $T_{P}\left(\Phi_{P},p_{P}\right)$ is given by

(2) $$T_{P}\left(\Phi_{P},p_{P}\right)=\left[\begin{array}{cc} R_{P}\left(\Phi_{P}\right) & p_{P}\\ 0_{1\times 3} & 1 \end{array}\right],$$

where

(3) $$R_{P}\left(\Phi_{P}\right)=\left[\begin{array}{ccc} c(\psi )c(\phi ) & c(\psi )s(\phi )s(\theta )-s(\psi )c(\theta ) & c(\psi )s(\phi )c(\theta )+s(\psi )s(\theta )\\ s(\psi )c(\phi ) & s(\psi )s(\phi )s(\theta )+c(\psi )c(\theta ) & s(\psi )s(\phi )s(\theta )-c(\psi )s(\theta )\\ -s(\phi ) & c(\phi )s(\theta ) & c(\phi )c(\theta ) \end{array}\right].$$

Here, $c\left(\;\cdot \;\right)$ is the cosine function and $s\left(\;\cdot \;\right)$ is the sine function. Note, $T_{P}\left(\;\cdot \;,\;\cdot \;\right)$ transforms points in Frame *P* into Frame *G*; equally, it describes the location of Frame *P* in Frame *G*. The top left 3×3 block of the $T_{P}\left(\;\cdot \;,\;\cdot \;\right)$ matrix is the rotation matrix which aligns Frame *P* in Frame *G*.

The IMU provides measurements at 50 Hz; the GNSS receivers are RTK-enabled and provide observations at 10 Hz. The LiDAR is a Velodyne VLP-16 and provides range measurements from 16 beams, equally spaced across a field of view of 30${}^{\circ }$ with 360${}^{\circ }$ and rotation at 10 Hz. Other details of the sensors are given in Table 1.

## 3. Pose Estimation Using GNSS, IMU, and LiDAR Measurements

**Figure 2:**
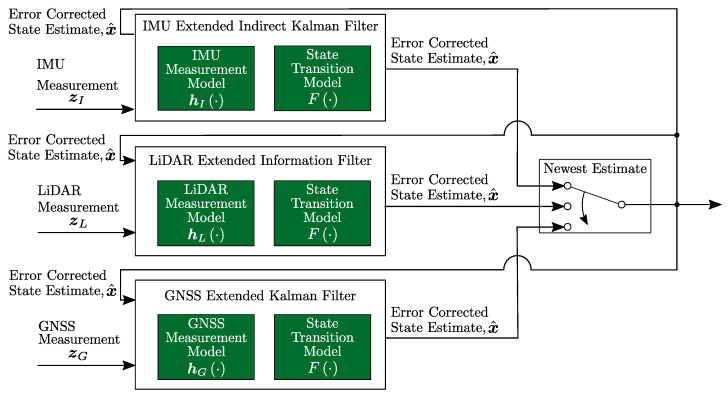
A diagrammatic representation of the proposed loosely coupled LiDAR- and GNSS-aided navigation system.

The pose estimator has the structure given in Figure 2. Each block corresponds to a Kalman filter update. On receipt of a sensor measurement, a filter update occurs that depends on the type of the measurement (IMU, GNSS, LiDAR) with the structure designed to accommodate different data rates, including, prospectively, sequences of lost measurements. The structure takes advantage of the high-frequency IMU measurements, updated through an indirect Kalman filter, and the low-frequency GNSS and LiDAR measurements, which are updated through Kalman and Information filters, respectively. The structure depicted in Figure 2 is generally known as an indirect, or error-state, Kalman filter. It is the common method for implementing IMU-aided navigation solutions [42,43,44,45,46,47,48,49,50]. The formulation below assumes that IMU data arrive at a higher rate than either GNSS or LiDAR measurements.

Algorithm 1 gives the algorithm based on updates from the IMU, GNSS, and LiDAR measurements.
**Algorithm 1:** LiDAR- and GNSS aided IMU navigation solution algorithm.
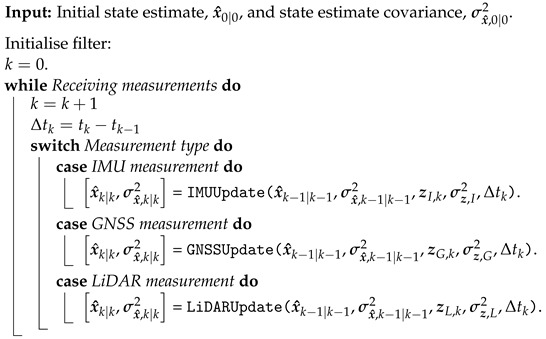


## 4. Process Model

The three filter updates of Figure 2 share the same process model which is assumed linear having the discrete-time form

(4) $$x_{k+1}=F\left(t_{k+1}-t_{k}\right)\;\cdot \;x_{k}+\left[\int_{t_{k}}^{t_{k+1}}F\left(t_{k+1}-\tau \right)d\beta \left(\tau \right)\right].$$

The state transition model $F\left(\Delta t_{k+1}\right)=F\left(t_{k+1}-t_{k}\right)$ predicts the platform’s state vector assuming a constant acceleration for translations and a constant rate for rotations. The state transition model also predicts the IMU acceleration and rotational rate biases through use of constants determined from experimental observations (see [51]). The 21×21 matrix $F\left(\;\cdot \;\right)$ has the form

(5) $$F\left(\Delta t_{k}\right)=\left[\begin{array}{ccccccc} I_{3\times 3} & \Delta t_{k}\;\cdot \;I_{3\times 3} & \frac{\Delta t_{k}^{2}}{2}\;\cdot \;I_{3\times 3} & 0_{3\times 3} & 0_{3\times 3} & 0_{3\times 3} & 0_{3\times 3}\\ 0_{3\times 3} & I_{3\times 3} & \Delta t_{k}\;\cdot \;I_{3\times 3} & 0_{3\times 3} & 0_{3\times 3} & 0_{3\times 3} & 0_{3\times 3}\\ 0_{3\times 3} & 0_{3\times 3} & I_{3\times 3} & 0_{3\times 3} & 0_{3\times 3} & 0_{3\times 3} & 0_{3\times 3}\\ 0_{3\times 3} & 0_{3\times 3} & 0_{3\times 3} & I_{3\times 3} & \Delta t_{k}\;\cdot \;I_{3\times 3} & 0_{3\times 3} & 0_{3\times 3}\\ 0_{3\times 3} & 0_{3\times 3} & 0_{3\times 3} & 0_{3\times 3} & I_{3\times 3} & 0_{3\times 3} & 0_{3\times 3}\\ 0_{3\times 3} & 0_{3\times 3} & 0_{3\times 3} & 0_{3\times 3} & 0_{3\times 3} & \frac{T_{b_{\ddot{x},\ddot{y},\ddot{z}}}}{T_{b_{\ddot{x},\ddot{y},\ddot{z}}}+\Delta t_{k}}\;\cdot \;I_{3\times 3} & 0_{3\times 3}\\ 0_{3\times 3} & 0_{3\times 3} & 0_{3\times 3} & 0_{3\times 3} & 0_{3\times 3} & 0_{3\times 3} & \frac{T_{b_{\omega_{x},\omega_{y},\omega_{z}}}}{T_{b_{\omega_{x},\omega_{y},\omega_{z}}}+\Delta t_{k}}\;\cdot \;I_{3\times 3} \end{array}\right].$$

Here, $T_{b_{\ddot{x},\ddot{y},\ddot{z}}}$ describes the empirically determined accelerometer bias parameters, and $T_{b_{\omega_{x},\omega_{y},\omega_{z}}}$ the empirically determined gyroscope bias parameters. Observe that

(6) $$\lim_{\Delta t_{k}\to 0}F\left(\Delta t\right)=I_{21\times 21}.$$

The second term of the right-hand side of Equation (4) describes the process noise which accounts for modelling approximations and model integration errors. We define

(7) $$w\left(t_{k}\right)=\left[\int_{t_{k}}^{t_{k+1}}F\left(t_{k+1}-\tau \right)d\beta \left(\tau \right)\right],$$

where $w\left(\;\cdot \;\right)$ is a white Gaussian discrete-time process and $\beta \left(\;\cdot \;\right)$ is Brownian motion with diffusion $\sigma_{Q}^{2}\left(t\right)$ such that for consecutive measurement times $t_{k}$ and $t_{k+1}$,

(8) $$\begin{array}{cc} E\left[\beta \left(t_{k}\right)-\beta \left(t_{k+1}\right)\right] & =0, \end{array}$$

(9) $$\begin{array}{cc} E\left[\left\{\beta \left(t_{k}\right)-\beta \left(t_{k+1}\right)\right\}{\left\{\beta \left(t_{k}\right)-\beta \left(t_{k+1}\right)\right\}}^{T}\right] & =\int_{t_{k}}^{t_{k+1}}\sigma_{Q}^{2}\left(t\right)dt. \end{array}$$

If the sample period is assumed to be small compared to the auto-correlation times of the process model modes,

(10) $$\sigma_{Q,k+1}^{2}=E\left[w\left(t_{k}\right)w^{T}\left(t_{k}\right)\right]\approx \sigma_{Q}^{2}\left(t_{k}\right)\;\cdot \;\left[t_{k+1}-t_{k}\right].$$

Observe that if the diffusion $\sigma_{Q}^{2}\left(t\right)$ is time-invariant, the process noise covariance, $\sigma^{2}$, increases linearly from the time since the last measurement. As this time increases, the process noise increases, resulting in the process model being weighted less in the Kalman filter update and measurements being weighted more.

## 5. Pose State Updates from IMU Measurements

### 5.1. IMU Measurement Model

**Figure 3:**
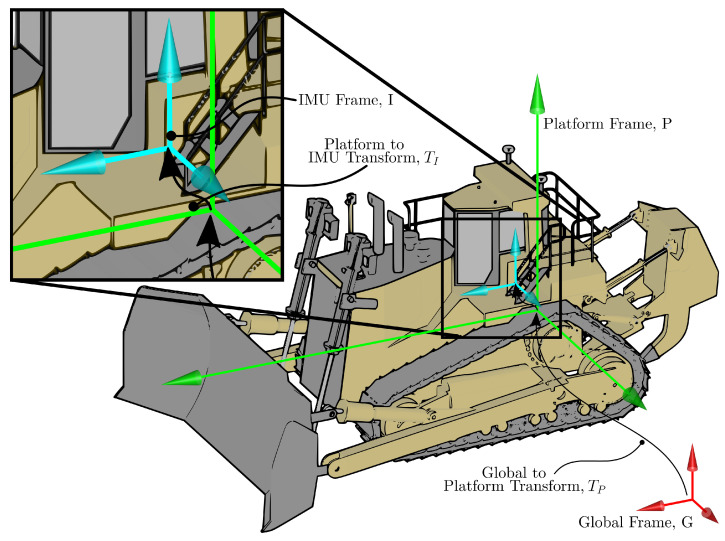
IMU Frame, *I*, relative to the platform Frame, *P*, and the global frame, *G*.

The IMU is installed on the platform frame close to the platform’s centre of gravity and is defined by Frame *I*—see Figure 3. IMU measurements are made in an inertial frame instantaneously aligned with Frame *I*. The IMU measurement model follows from Equation (3) and takes the form

(11) $$z_{I}=h_{I}\left(x\right)+v_{z,I},$$

where

(12) $$z_{I}={\left[\begin{array}{cc} a_{I} & \omega_{I} \end{array}\right]}^{T}={\left[\begin{array}{cccccc} {\ddot{x}}_{I} & {\ddot{y}}_{I} & {\ddot{z}}_{I} & {\dot{\theta }}_{I} & {\dot{\phi }}_{I} & {\dot{\psi }}_{I} \end{array}\right]}^{T}$$

and measurement noise $v_{z,I}$ is assumed to be white and Gaussian with covariance $\sigma_{z,I}^{2}$—see Table 2.

To determine the function $h_{I}\left(\;\cdot \;\right)$, note that the position of the origin of the IMU in the global frame is given by

(13) $$\left[\begin{array}{c} p_{I}\left(t\right)\\ 1 \end{array}\right]=\left[\begin{array}{cc} R_{P}\left(t\right) & p_{P}\left(t\right)\\ 0_{1\times 3} & 1 \end{array}\right]\left[\begin{array}{c} p_{i}\\ 1 \end{array}\right],$$

where $p_{i}$ is the (fixed and known) position of the origin of Frame *I* in Frame *P*. The velocity of this point at time *t* is

(14) $$\left[\begin{array}{c} {\dot{p}}_{I}\left(t\right)\\ 0 \end{array}\right]=\left[\begin{array}{cc} {\dot{R}}_{P}\left(t\right) & {\dot{p}}_{P}\left(t\right)\\ 0_{1\times 3} & 0 \end{array}\right]\left[\begin{array}{c} p_{i}\\ 1 \end{array}\right]=\left[\begin{array}{cc} \Omega_{P}\left(t\right) & v_{P}\left(t\right)\\ 0_{1\times 3} & 1 \end{array}\right]\left[\begin{array}{c} p_{I}\left(t\right)\\ 1 \end{array}\right],$$

where $\Omega_{P}\left(t\right)$ is the anti-symmetric matrix representing the angular velocity of Frame *P* in Frame *G*. The matrix $\Omega_{P}\left(t\right)$ is

(15) $$\Omega_{P}=\mathrm{sk}\left(\omega_{P}\right)=\left[\begin{array}{ccc} 0 & -\omega_{z} & \omega_{y}\\ \omega_{z} & 0 & -\omega_{x}\\ -\omega_{y} & \omega_{x} & 0 \end{array}\right].$$

The acceleration of the origin of Frame *I* in Frame *G* is found by differentiating Equation (14), i.e.,

(16) $$\left[\begin{array}{c} {\ddot{p}}_{I}\\ 0 \end{array}\right]=\left[\begin{array}{cc} {\ddot{R}}_{P}\left(t\right) & {\ddot{p}}_{P}\left(t\right)\\ 0_{1\times 3} & 0 \end{array}\right]\left[\begin{array}{c} p_{i}\\ 1 \end{array}\right].$$

Noting that if a constant angular rate is assumed (i.e., ${\dot{\Omega }}_{P}=0$),

(17) $${\ddot{R}}_{P}\left(t\right)={\dot{\Omega }}_{P}\left(t\right)\;\cdot \;R_{P}\left(t\right)+\Omega_{P}\left(t\right)\;\cdot \;{\dot{R}}_{P}\left(t\right)={\dot{\Omega }}_{P}\left(t\right)\;\cdot \;R_{P}\left(t\right)+\Omega_{P}^{2}\left(t\right)\;\cdot \;R_{P}\left(t\right)=\Omega_{P}^{2}\left(t\right)\;\cdot \;R_{P}\left(t\right),$$

which gives,

(18) $$\left[\begin{array}{c} {\ddot{p}}_{I}\left(t\right)\\ 0 \end{array}\right]=\left[\begin{array}{cc} \Omega_{P}^{2}\left(t\right)\;\cdot \;R_{P}\left(t\right) & {\ddot{p}}_{P}\left(t\right)\\ 0_{1\times 3} & 0 \end{array}\right]\left[\begin{array}{c} p_{i}\left(t\right)\\ 1 \end{array}\right].$$

The measurement equations follow from Equation (18) and can be expressed in terms of the pose state vector:

(19) $$z_{I}=h_{I}\left(x\right)=\left[\begin{array}{c} a_{I}\\ \omega_{I} \end{array}\right]=R_{I}^{T}\;\cdot \;R_{P}^{T}\left(\Phi_{P}\right)\;\cdot \;\left[\begin{array}{c} \Omega_{P}^{2}\left(\omega_{P}\right)\;\cdot \;R_{P}\left(\Phi_{P}\right)\;\cdot \;p_{i}+{\ddot{p}}_{P}+g\\ \omega_{P} \end{array}\right]+\left[\begin{array}{c} b_{a}\\ b_{r} \end{array}\right].$$

Note that the rotation matrix $R_{I}^{T}\;\cdot \;R_{P}^{T}\left(\;\cdot \;\right)$ serves to align the acceleration and rate vectors to Frame *I* in which measurements are made. The vector $b_{r}$ is the bias of rate gyro, $b_{a}$ is the accelerometer bias, and g denotes gravitational acceleration.

The Jacobian of $h\left(\;\cdot \;\right)$ can be found analytically from

(20) $$\nabla h_{I}\left(x\right)=\left[\begin{array}{ccccccc} 0_{3\times 3} & 0_{3\times 3} & I_{3\times 3} & M_{1} & M_{2} & I_{3\times 3} & 0_{3\times 3}\\ 0_{3\times 3} & 0_{3\times 3} & 0_{3\times 3} & R_{I}^{T}\;\cdot \;\frac{\partial R_{P}\left(\Phi_{P}\right)}{\partial \Phi_{P}}\;\cdot \;\omega_{P} & R_{I}^{T}\;\cdot \;R_{P}^{T}\left(\Phi_{P}\right) & 0_{3\times 3} & I_{3\times 3} \end{array}\right],$$

where

(21) $$\begin{array}{c} \begin{array}{cc} M_{1} & =R_{I}^{T}\;\cdot \;\frac{\partial R_{P}^{T}\left(\Phi_{P}\right)}{\partial \Phi_{P}}\;\cdot \;\left(\Omega_{P}^{2}\left(\omega_{P}\right)\;\cdot \;R_{P}\left(\Phi_{P}\right)\;\cdot \;p_{i}+{\ddot{p}}_{P}+g\right)\\ \; & \;+R_{I}^{T}\;\cdot \;R_{P}^{T}\left(\Phi_{P}\right)\;\cdot \;\Omega_{P}^{2}\left(\omega_{P}\right)\;\cdot \;\frac{\partial R_{P}\left(\Phi_{P}\right)}{\partial \Phi_{P}}\;\cdot \;p_{i}, \end{array} \end{array}$$

(22) $$\begin{array}{cc} M_{2} & =R_{I}^{T}\;\cdot \;R_{P}^{T}\left(\Phi_{P}\right)\;\cdot \;\frac{\partial \Omega_{P}^{2}\left(\omega_{P}\right)}{\partial \omega_{P}}\;\cdot \;R_{P}\left(\Phi_{P}\right)\;\cdot \;p_{i}. \end{array}$$

### 5.2. IMU Updates

The approach to updating measurements from the IMU involves estimating the error on the state estimate, $\delta {\hat{x}}_{k|k}^{\prime }$, at time $t_{k}$ given measurements to time $t_{k}$, and combining this with the propagated state estimate, ${\hat{x}}_{k|k}^{\prime }$, from the most recent GNSS or LiDAR update. The state estimate update, ${\hat{x}}_{k|k}$, is then computed as the sum,

(23) $${\hat{x}}_{k|k}={\hat{x}}_{k|k}^{\prime }+\delta {\hat{x}}_{k|k}^{\prime }.$$

The IMU filter is reset after each GNSS or LiDAR update: state estimate and state estimate covariance are set from the error-corrected state estimate and error-corrected state estimate covariance,

(24) $$\begin{array}{cc} {\hat{x}}_{k-1|k-1}^{\prime } & ={\hat{x}}_{k-1|k-1}, \end{array}$$

(25) $$\begin{array}{cc} \sigma_{{\hat{x}}^{\prime },k-1|k-1}^{2} & =\sigma_{\hat{x},k-1|k-1}^{2}, \end{array}$$

and the error state estimate and error state estimate covariance are initialised to zero:

(26) $$\begin{array}{cc} \delta {\hat{x}}_{k-1|k-1}^{\prime } & =0_{21\times 1}, \end{array}$$

(27) $$\begin{array}{cc} \sigma_{\delta {\hat{x}}^{\prime },k-1|k-1}^{2} & =0_{21\times 21}. \end{array}$$

On receipt of an IMU measurement, the calculation of $\delta {\hat{x}}_{k|k}$ starts with the prediction of the state estimate,

(28) $${\hat{x}}_{k|k-1}^{\prime }=F\left(\Delta t_{k}\right)\;\cdot \;{\hat{x}}_{k-1|k-1}^{\prime },$$

the error state,

(29) $$\delta {\hat{x}}_{k|k-1}^{\prime }=F\left(\Delta t_{k}\right)\;\cdot \;\delta {\hat{x}}_{k-1|k-1}^{\prime },$$

state covariance,

(30) $$\sigma_{{\hat{x}}^{\prime },k|k-1}^{2}=F\left(\Delta t_{k}\right)\;\cdot \;\sigma_{{\hat{x}}^{\prime },k-1|k-1}^{2}\;\cdot \;F{\left(\Delta t_{k}\right)}^{T}.$$

and error state covariance,

(31) $$\sigma_{\delta {\hat{x}}^{\prime },k|k-1}^{2}=F\left(\Delta t_{k}\right)\;\cdot \;\sigma_{\delta {\hat{x}}^{\prime },k-1|k-1}^{2}\;\cdot \;F{\left(\Delta t_{k}\right)}^{T}+\sigma_{Q,k}^{2}.$$

The innovation is computed from

(32) $$\nu_{I,k}=z_{I,k}-\left(h_{I}\left({\hat{x}}_{k|k-1}^{\prime }\right)+\nabla h_{I}\left({\hat{x}}_{k|k-1}^{\prime }\right)\;\cdot \;\delta {\hat{x}}_{k|k-1}^{\prime }\right)=z_{I,k}-\left({\hat{z}}_{I,{\hat{x}}_{k|k-1}^{\prime }}+{\hat{z}}_{I,\delta {\hat{x}}_{k|k-1}^{\prime }}\right),$$

and the innovation covariance, $\sigma_{\nu ,k}^{2}$, and Kalman gain, $W_{I,k}$, are found from

(33) $$\begin{array}{cc} \sigma_{\nu ,I,k}^{2} & =\nabla h_{I}\left({\hat{x}}_{k|k-1}^{\prime }\right)\;\cdot \;\sigma_{\delta {\hat{x}}^{\prime },k|k-1}^{2}\;\cdot \;\nabla h_{I}{\left({\hat{x}}_{k|k-1}^{\prime }\right)}^{T}+\sigma_{z,I}^{2}, \end{array}$$

(34) $$\begin{array}{cc} W_{I,k} & =\sigma_{\delta {\hat{x}}^{\prime },k|k-1}^{2}\;\cdot \;\nabla h_{I}{\left({\hat{x}}_{k|k-1}^{\prime }\right)}^{T}\;\cdot \;{\left(\sigma_{\nu ,I,k}^{2}\right)}^{-1}. \end{array}$$

The corrected error state and the corrected error state covariance are computed from

(35) $$\begin{array}{cc} \delta {\hat{x}}_{k|k}^{\prime } & =\delta {\hat{x}}_{k|k-1}^{\prime }+W_{I,k}\;\cdot \;\nu_{I,k}, \end{array}$$

(36) $$\begin{array}{cc} \sigma_{\delta {\hat{x}}^{\prime },k|k}^{2} & =\sigma_{\delta {\hat{x}}^{\prime },k|k-1}^{2}-W_{I,k}\;\cdot \;\sigma_{\nu ,I,k}^{2}\;\cdot \;W_{I,k}^{T}. \end{array}$$

The predicted but uncorrected state estimate, ${\hat{x}}_{k|k-1}$, and state estimate covariance, $\sigma_{\hat{x},k|k-1}^{2}$, are used in the next iteration of the filter,

(37) $$\begin{array}{cc} {\hat{x}}_{k|k}^{\prime } & ={\hat{x}}_{k|k-1}^{\prime }, \end{array}$$

(38) $$\begin{array}{cc} \sigma_{{\hat{x}}^{\prime },k|k}^{2} & =\sigma_{{\hat{x}}^{\prime },k|k-1}^{2}. \end{array}$$

The error-corrected state estimate, ${\hat{x}}_{k|k}$, which incorporates the newest IMU observation, is computed through the addition with the error state estimate,

(39) $$\begin{array}{cc} {\hat{x}}_{k|k} & ={\hat{x}}_{k|k}^{\prime }+\delta {\hat{x}}_{k|k}^{\prime }, \end{array}$$

(40) $$\begin{array}{cc} \sigma_{\hat{x},k|k}^{2} & =\sigma_{{\hat{x}}^{\prime },k|k}^{2}+\sigma_{\delta {\hat{x}}^{\prime },k|k}^{2}. \end{array}$$

The indirect extended Kalman filter (IEKF) algorithm for IMU updates is detailed in Algorithm 2.    
**Algorithm 2:** IMUUpdate - An Indirect Extended Kalman Filter (IEKF) algorithm for state estimation using IMU observations.
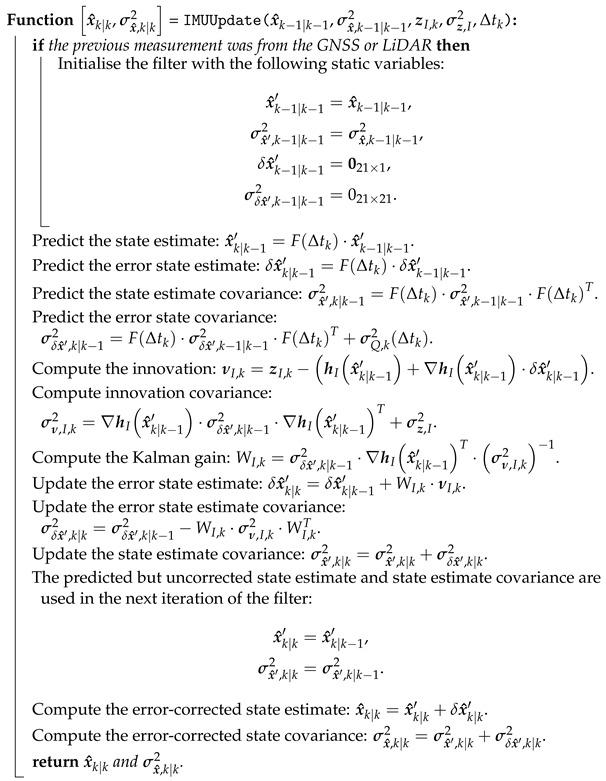


## 6. Pose State Updates from GNSS Measurements

### 6.1. GNSS Measurement Model

The GNSS measurement model gives the locations of the GNSS receivers in the global frame and takes the form

(41) $$z_{G}=h_{G}\left(x\right)+v_{z,G},$$

**Figure 4:**
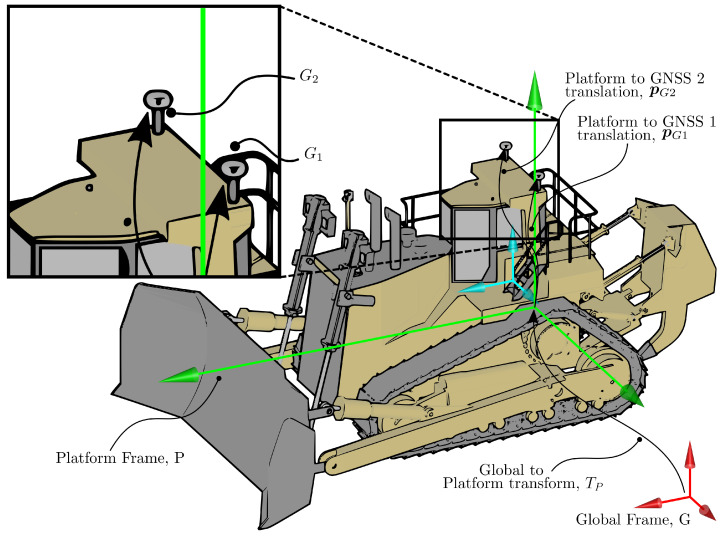
The locations of GNSS 1, $p_{G2}$, from the platform frame, *P*.

Here, $h_{G}\left(\;\cdot \;\right)$ gives the locations of the GNSS receivers as a function of the state,

(42) $$z_{G}=h_{G}\left(x\right)=\left[\begin{array}{c} R_{P}\left(\Phi_{P}\right)\;\cdot \;p_{G1}+p_{P}\\ R_{P}\left(\Phi_{P}\right)\;\cdot \;p_{G2}+p_{P} \end{array}\right],$$

where $p_{G1}$ and $p_{G2}$ are the coordinates of the GNSS antennas in the platform frame (see Figure 4). The measurement vector $z_{G}$ is the stacked vectors of the antennas’ Cartesian coordinates in Frame *G*. Equation (42) can be readily extended if a third GNSS receiver were mounted to the platform. The measurement noise, $v_{z,G}$, is assumed to be white and Gaussian with covariance $\sigma_{z,G}^{2}$ (see Table 3).

The GNSS measurement Jacobian is given by

(43) $$\nabla h_{G}\left(x\right)=\left[\begin{array}{ccccccc} I_{3\times 3} & 0_{3\times 3} & 0_{3\times 3} & \frac{\partial R_{P}^{T}\left(\Phi_{P}\right)}{\partial \Phi_{P}}\;\cdot \;p_{G1} & 0_{3\times 3} & 0_{3\times 3} & 0_{3\times 3}\\ I_{3\times 3} & 0_{3\times 3} & 0_{3\times 3} & \frac{\partial R_{P}^{T}\left(\Phi_{P}\right)}{\partial \Phi_{P}}\;\cdot \;p_{G2} & 0_{3\times 3} & 0_{3\times 3} & 0_{3\times 3} \end{array}\right].$$

### 6.2. GNSS Updates

The estimation of the error-corrected state estimate, ${\hat{x}}_{k|k}$, begins with a prediction of state using a state transition model, $F\left(\Delta t_{k}\right)$, and the previous error-corrected state estimate, ${\hat{x}}_{k-1|k-1}$,

(44) $${\hat{x}}_{k|k-1}=F\left(\Delta t_{k}\right)\;\cdot \;\left({\hat{x}}_{k-1|k-1}\right),$$

and the prediction of the state covariance,

(45) $$\sigma_{\hat{x},k|k-1}^{2}=F\left(\Delta t_{k}\right)\;\cdot \;\sigma_{\hat{x},k-1|k-1}^{2}\;\cdot \;F{\left(\Delta t_{k}\right)}^{T}+\sigma_{Q,k}^{2}.$$

The GNSS measurement innovation and innovation covariance are computed from

(46) $$\begin{array}{cc} \nu_{G,k} & =z_{G,k}-h_{G}\left({\hat{x}}_{k|k-1}\right)=z_{G,k}-{\hat{z}}_{G,k}, \end{array}$$

(47) $$\begin{array}{cc} \sigma_{\nu ,G,k}^{2} & =\nabla h_{G}\left({\hat{x}}_{k|k-1}\right)\;\cdot \;\sigma_{\hat{x},k|k-1}^{2}\;\cdot \;\nabla h_{G}{\left({\hat{x}}_{k|k-1}\right)}^{T}+\sigma_{z,G}^{2}, \end{array}$$

where $z_{G,k}$ is the GNSS measurement.

The Kalman gain, $W_{G,k}$, is then computed using

(48) $$W_{G,k}=\sigma_{\hat{x},k|k-1}^{2}\;\cdot \;\nabla h_{G}{\left({\hat{x}}_{k|k-1}\right)}^{T}\;\cdot \;{\left(\sigma_{\nu ,G,k}^{2}\right)}^{-1}.$$

The error-corrected state estimate, ${\hat{x}}_{k|k}$, and error-corrected state estimate covariance, $\sigma_{\hat{x},k|k}^{2}$, are computed using

(49) $$\begin{array}{cc} {\hat{x}}_{k|k} & ={\hat{x}}_{k|k-1}+W_{G,k}\;\cdot \;\nu_{G,k}, \end{array}$$

(50) $$\begin{array}{cc} \sigma_{\hat{x},k|k}^{2} & =\sigma_{\hat{x},k|k-1}^{2}-W_{G,k}\;\cdot \;\sigma_{\nu ,G,k}^{2}\;\cdot \;W_{G,k}^{T}. \end{array}$$

The GNSS update is given as Algorithm 3.    
**Algorithm 3:** GNSSUpdate—An extended Kalman filter (EKF) algorithm for state estimation using GNSS observations.
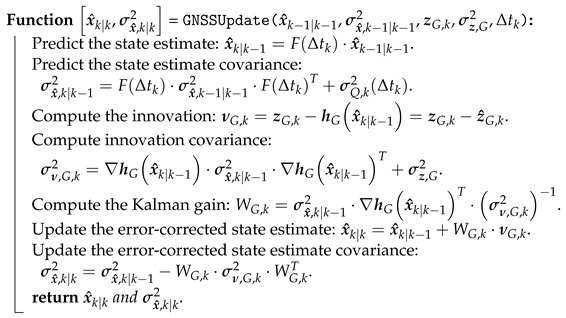


## 7. Pose State Updates Using LiDAR Measurements

### 7.1. LiDAR Measurement Model in a Known Environment

The LiDAR measurement model developed in this section computes the expected range measurements based on the state vector and a triangulated model of the environment, denoted *X*. The LiDAR measurement model ray-casts from the LiDAR to the environment model *X* to determine the expected measurement ranges from the LiDAR sensor. In this section, the terrain model *X* is assumed known; in Section 9, the approach is generalised to unknown terrain.

The LiDAR measurement function is denoted

(51) $$z_{L}=h_{L}\left(x,X\right)+v_{z,L},$$

where the LiDAR measurement vector, $z_{L}$ is a set of ranges corresponding to each ray from the LiDAR sensor. For *n* ranges,

(52) $$z_{L}={\left[\begin{array}{cccc} z_{0} & z_{1} & \cdots & z_{n} \end{array}\right]}^{T}.$$

The measurement noise $v_{z,L}$ is assumed to be white and Gaussian with covariance $\sigma_{z,L}^{2}$—see Section 7.2.

**Figure 5:**
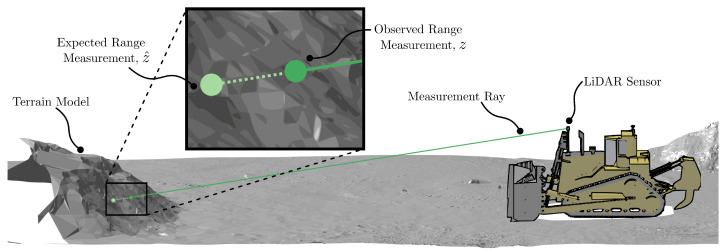
The LiDAR measurement model provides the measurement that the LiDAR sensor is expected to observe given the current state vector. The difference between the expected and observed measurements is termed the innovation.

The LiDAR measurement function, $h_{L}\left(\;\cdot \;,\;\cdot \;\right)$, determines the expected sensor observations by ray-casting against a known terrain model given a pose state estimate, *X* (see Figure 5). Each ray emanates from a point $s_{0}$ in Frame *L* known as the sensor origin. The direction of each ray or beam of the LiDAR can be defined by a second point $s_{j}$, j=1…n with $\left|\right|s_{j}-s_{0}\left|\right|=1$. The location of these points in Frame *G* can be found with transformations

(53) $$\begin{array}{cc} q_{0} & =T_{P}\left(\Phi_{P},p_{P}\right)\;\cdot \;T_{L}\;\cdot \;s_{0}, \end{array}$$

(54) $$\begin{array}{cc} q_{j} & =T_{P}\left(\Phi_{P},p_{P}\right)\;\cdot \;T_{L}\;\cdot \;s_{j}. \end{array}$$

Here, $T_{L}$ describes the location of Frame *L* in Frame *P*. This transform is assumed known. See [52] for approaches to finding it.

Each ray is parametrised by a range $r_{j}$ and can be described by

(55) $$\Gamma \left(r_{j}\right)=q_{0}+r_{j}\;\cdot \;\left(q_{j}-q_{0}\right).$$

To find $r_{j}$, it is necessary to first determine the triangle in *X* which it intersects. Denote the vertices of this triangle, $\Delta_{j}=\left(\alpha_{j},\beta_{j},\gamma_{j}\right)$. Then,

(56) $$r_{j}=\frac{n_{j}\;\cdot \;\left(\alpha_{j}-q_{0}\right)}{n_{j}\;\cdot \;\left(q_{0}-q_{j}\right)},$$

where

(57) $$n_{j}=\left(\beta_{j}-\alpha_{j}\right)\times \left(\gamma_{j}-\alpha_{j}\right).$$

**Figure 6:**
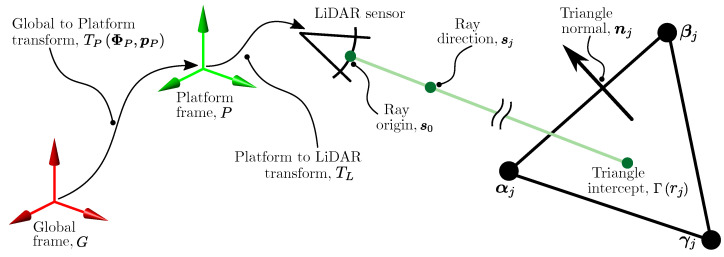
The measurement model for the LiDAR calculates the expected range measurement as the distance from the ray origin to the intercepted triangle of the model.

The ray-triangle intersection is depicted in Figure 6.

The measurement model for *n* ray intersections can be constructed from

(58) $$z_{L}={\left[r_{1},r_{2},\ldots ,r_{n}\right]}^{T},$$

and the LiDAR measurement Jacobian from

(59) $$\nabla h_{L}\left(x,X\right)=\left[\begin{array}{cccc} \frac{\partial r_{1}}{\partial p_{P}} & 0_{1\times 6} & \frac{\partial r_{1}}{\partial \Phi_{P}} & 0_{1\times 9}\\ \frac{\partial r_{2}}{\partial p_{P}} & 0_{1\times 6} & \frac{\partial r_{2}}{\partial \Phi_{P}} & 0_{1\times 9}\\ \vdots & \vdots & \vdots & \vdots \\ \frac{\partial r_{n}}{\partial p_{P}} & 0_{1\times 6} & \frac{\partial r_{n}}{\partial \Phi_{P}} & 0_{1\times 9} \end{array}\right].$$

### 7.2. LiDAR Measurement Noise Model

Observations from the LiDAR sensor present in this application are transformed from the LiDAR frame, *L*, into the global frame, *G*. However, each of the transforms are required to achieve this contain uncertainty. For sensors such as the GNSS receivers and IMU, the uncertainty introduced by these transforms is small, as observations are obtained at the sensor. However, for perception sensors such as LiDAR, which perceive the environment at potentially significant ranges, small transform errors can lead to quite significant observation errors. For example, a registration error of 1${}^{\circ }$ at 50 m results in ∼ 0.7 m of endpoint error.

**Figure 7:**
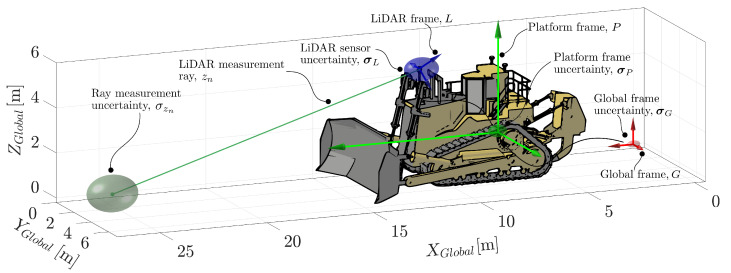
The location of each of the frames of the platform are uncertain to varying degrees. This uncertainty is propagated through the transforms of the system, originating at the global frame and ending at the ray endpoint. The propagated uncertainty is the effective uncertainty of that ray’s endpoint.

The uncertainty of frame locations and measurements may be propagated from their origin to the point of measurement to determine the effective uncertainty. We consider four sources of uncertainty which we propagate from their origin to the LiDAR ray endpoint (shown in Figure 7). The sources of uncertainty considered are (i) the uncertainty in the global frame location, $\sigma_{G}^{2}$; (ii) the estimated location of the platform relative to the global frame, $\sigma_{P}^{2}$; (iii) the uncertainty in the LiDAR registration, $\sigma_{L}^{2}$; and (iv) the LiDAR’s measurement uncertainty, $\sigma_{z,L}^{2}$. The Jacobian of each of the relevant transforms is used to transform the uncertainty through the system.

The uncertainty from each of these sources propagates through to the endpoint of the ray as follows:

(60) $$\begin{array}{cc} \sigma_{P,k}^{2} & =\nabla T_{P}\left(\Phi_{P},p_{P}\right)\;\cdot \;\sigma_{G}^{2}\;\cdot \;\nabla T_{P}{\left(\Phi_{P},p_{P}\right)}^{T}+\sigma_{P}^{2}, \end{array}$$

(61) $$\begin{array}{cc} \sigma_{L,k}^{2} & =\nabla T_{L}\;\cdot \;\sigma_{P,k}^{2}\;\cdot \;\nabla T_{L}^{T}+\sigma_{L}^{2}, \end{array}$$

(62) $$\begin{array}{cc} \sigma_{z,L}^{2} & =\nabla T_{R}\;\cdot \;\sigma_{L,k}^{2}\;\cdot \;\nabla T_{R}^{T}+\sigma_{z,L}^{2}. \end{array}$$

Here, the notation $\nabla T_{L}$ is used to denote the Jacobian of the homogeneous transform—in this case, the transform from the platform frame to the LiDAR frame.

Figure 7 provides a visual representation of the propagation of uncertainty from the global frame through to the endpoint of the ray. The covariances shown in Figure 7 have been exaggerated for visual clarity.

### 7.3. LiDAR Updates

The number of LiDAR measurements typically exceeds the dimension of the state vector so it is computationally more efficient to use the information form of the extended Kalman filter to perform the pose update from LiDAR measurements [53].

The current state and covariance are transformed in the information space

(63) $$\begin{array}{cc} Y_{k-1|k-1} & ={\left(\sigma_{\hat{x},k-1|k-1}^{2}\right)}^{-1}, \end{array}$$

(64) $$\begin{array}{cc} {\hat{y}}_{k-1|k-1} & =Y_{k-1|k-1}\;\cdot \;{\hat{x}}_{k-1|k-1}. \end{array}$$

The Fisher information matrix (FIM) and Fisher information vector (FIV) are predicted to the current time step using

(65) $$\begin{array}{cc} Y_{k|k-1} & ={\left[F\left(\Delta t_{k}\right)\;\cdot \;{\left(Y_{k-1|k-1}\right)}^{-1}\;\cdot \;F{\left(\Delta t_{k}\right)}^{T}+\sigma_{Q,k}^{2}\right]}^{-1}, \end{array}$$

(66) $$\begin{array}{cc} {\hat{y}}_{k|k-1} & =Y_{k|k-1}\;\cdot \;F\left(\Delta t_{k}\right)\;\cdot \;{\hat{x}}_{k-1|k-1}. \end{array}$$

The predicted state estimate is obtained from

(67) $$\begin{array}{c} {\hat{x}}_{k|k-1}={\left(Y_{k-1|k-1}\right)}^{-1}\;\cdot \;{\hat{y}}_{k|k-1} \end{array}$$

and the innovation is obtained using the LiDAR observation function, the current predicted state estimate, and the terrain model, *X*:

(68) $$\begin{array}{c} \nu_{L,k}=z_{L,k}-h_{L}\left({\hat{x}}_{k|k-1},X\right). \end{array}$$

The predicted FIV and FIM are corrected through use of the innovation and the LiDAR range measurements using

(69) $$\begin{array}{cc} {\hat{y}}_{k|k} & ={\hat{y}}_{k|k-1}+\nabla h_{L}{\left({\hat{x}}_{k|k-1},X\right)}^{T}\;\cdot \;{\left(\sigma_{z,L}^{2}\right)}^{-1}\;\cdot \;\left[\nu_{L,k}+\nabla h_{L}\left({\hat{x}}_{k|k-1},X\right)\;\cdot \;{\hat{x}}_{k|k-1}\right], \end{array}$$

(70) $$\begin{array}{cc} Y_{k|k} & =Y_{k|k-1}+\nabla h_{L}{\left({\hat{x}}_{k|k-1},X\right)}^{T}\;\cdot \;{\left(\sigma_{z,L}^{2}\right)}^{-1}\;\cdot \;\nabla h_{L}\left({\hat{x}}_{k|k-1},X\right). \end{array}$$

The FIM and FIV are now transformed back into the state space,

(71) $$\begin{array}{cc} {\hat{x}}_{k|k} & ={\left(Y_{k|k}\right)}^{-1}\;\cdot \;{\hat{y}}_{k|k}, \end{array}$$

(72) $$\begin{array}{cc} \sigma_{\hat{x},k|k}^{2} & ={\left(Y_{k|k}\right)}^{-1}. \end{array}$$

Algorithm 4 shows the LiDAR pose estimation extended information filter (EIF) algorithmically.    
**Algorithm 4:** LiDARUpdate—An extended information filter (EIF) algorithm for state estimation using LiDAR observations in a known environment.
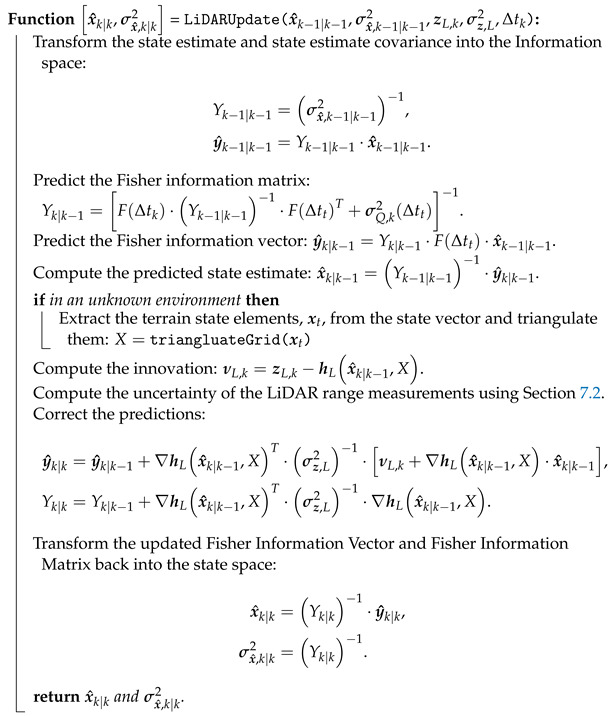


## 8. Evaluation

**Figure 8:**
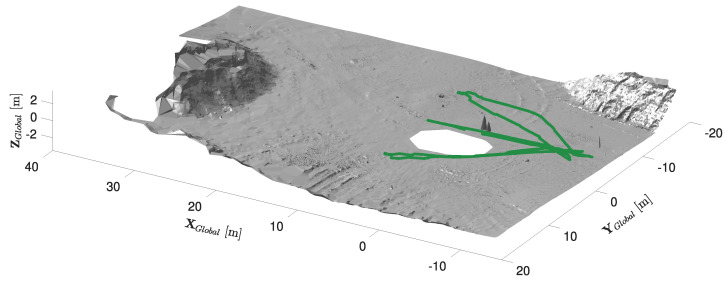
The environment in which the bulldozer platform manoeuvred is shown. The trajectory of the platform is shown as a green line. The platform exercised its rotational degrees of freedom through the use of the blade during this trajectory.

It is necessary to use indirect methods for the evaluation of the pose estimator as there no is ground truth pose trajectory. The approach chosen is to survey the environment to obtain a ground truth model and compare LiDAR measurements with the predicted ranges from ray-casting against this scanned ground truth model. These differences are representative of the error in the pose. The survey to obtain the ground truth model was created with a Faro Focus^3D^ terrestrial LiDAR scanner [54]. This instrument claims a one standard deviation range precision of 0.0011 m. Figure 8 shows the environment model obtained by scanning.

**Figure 9:**
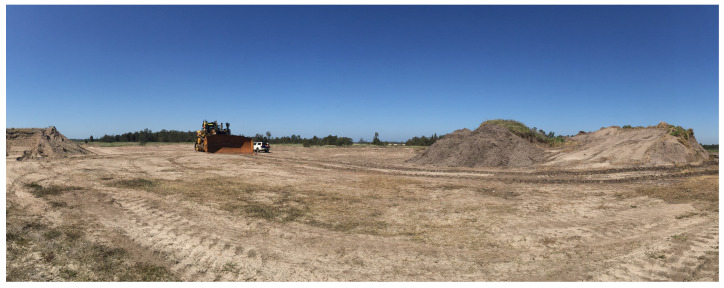
A photo of the environment in which the platform traversed is shown. Here, the bulldozer platform is visible in the centre of the image. The mound visible to the left of Figure 8 is visible to the right of this image.

The scene used for evaluation is shown in Figure 9. It comprises a generally flat terrain with piled dirt, forming mounds. The bulldozer was manually moved around in this environment following the path shown in Figure 8. During the execution of this motion, at various points, the platform was pitched and rolled using the bulldozer blade.

**Figure 10:**
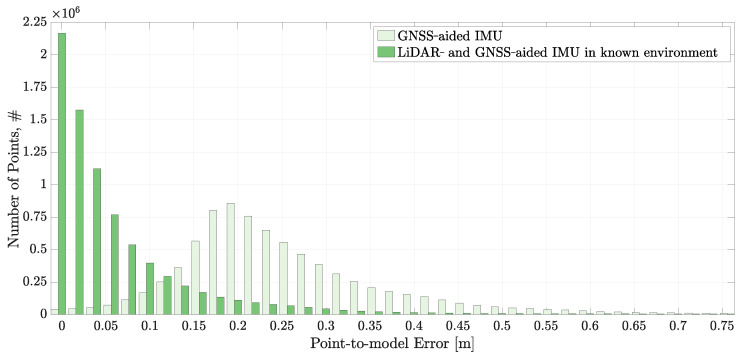
The point-to-model error distributions obtained by comparing the LiDAR endpoints with the ground truth terrain model using the GNSS-aided IMU and LiDAR- and GNSS-aided IMU navigation solutions.

Figure 10 shows the point-to-model errors for the GNSS-aided IMU solution and the LiDAR- and GNSS-aided solution in a known environment. A ray having the length of the measurement is cast along the corresponding LiDAR beam at the estimated pose. The distance from the end point of the ray to the nearest point of the model is determined as the point-to-model error. The smaller the point-to-model error, the closer the real pose to the estimated pose. For perfect pose, a perfect model, and zero measurement error, the point-to-model error is zero.

The distribution of point-to-model errors given in Figure 10 evidences that the LiDAR- and GNSS-aided IMU solution is more accurate than the GNSS aiding. The mean point-to-model error is reduced from 0.25 m to 0.06 m. Significantly, the highest number of point-to-model errors for the LiDAR- and GNSS-aided solution corresponds to zero error. This is not unexpected: the positioning of the GNSS antennas provides information that supports good spatial positioning along with roll and yaw estimation. However, no information is available from GNSS measurements about the orientation of the axis that passes through the two antenna centres. This rotation heavily contributes to the pitch of the platform. The provision of measurements of the forward-facing 3D LiDAR provides information that stabilises the pitch estimate, this accounting for the improved accuracy. The mean value for point-to-model error for the GNSS-aided solution is prospectively due to start-up bias on the centre. It is noted that the GNSS-aided solution has a broader distribution of error.

**Figure 11:**
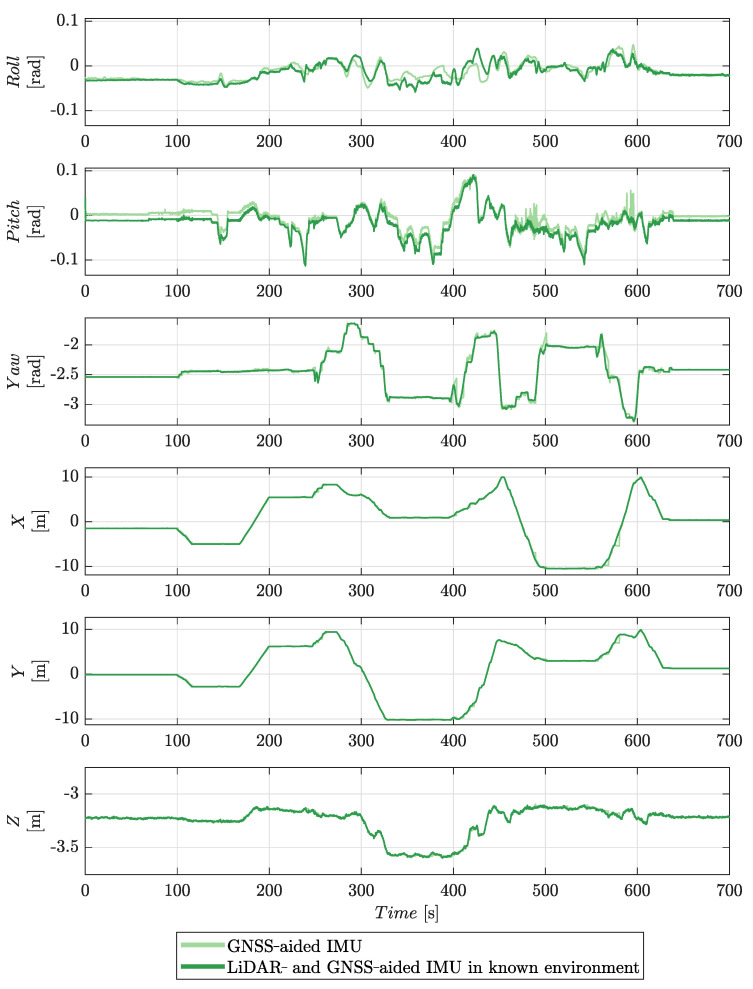
A comparison of the results from the GNSS-aided IMU and the LiDAR- and GNSS-aided IMU in a known environment navigation solutions.

The time estimates for the six degrees of freedom of the platform’s pose are shown in Figure 11. Significantly both estimators give similar results for *x*, *y*, *z*, and yaw. Most of the difference is in estimates for the pitch and roll of the platform. The bias in the pitch estimate for the GNSS-aided solution is attributed to start-up bias in the IMU sensor, which the estimator has been unable to account for; it is present at both the start and end of the motion sequence when the platform is stationary.

The results in this paper were produced on a computer running a Linux-based operating system (Intel Xeon W-2125 CPU, 32 GB of memory). The GNSS-aided IMU filter could update up to ∼ 10.000 Hz, much faster than the measurement rate. The LiDAR- and GNSS-aided IMU filter required more computing per time step and was capable of ∼ 500 Hz.

## 9. LiDAR Measurement Model in an Unknown Environment

The capability to estimate pose when the environment is known helps to establish the potential performance of the LiDAR- and GNSS-aiding estimator, but is of limited practical usefulness. Most environments of interest in which this platform operates have unknown geometry. The estimator is extended to achieve the benefits of pitch stabilisation provided by the LiDAR measurement by concurrently estimating the pose and the local terrain.

**Figure 12:**
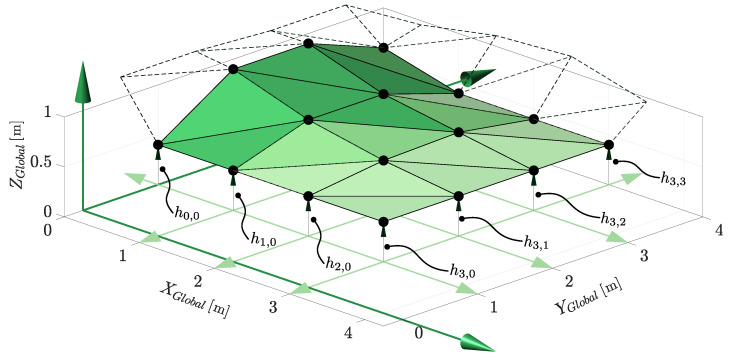
The terrain estimate is represented by a triangulated height grid structure. Each cell height is a state which we estimate in the filter.

For this purpose, the terrain is modelled as a height grid that divides the (x,y) plane into a regular grid. Each grid element, known as a cell, is assigned a height. The points defined by each cell in the height grid can be triangulated to develop a representation of the terrain as a surface. A representation of the triangulated height grid with states $h_{0,0}$ to $h_{3,3}$ is shown in Figure 12.

Each of the o·p=q cell heights in the grid can be considered additional states for which estimates are sought. These states, collectively denoted $x_{t}$, augment the state vector defined by Equation (1), shown here as $x_{P}$. Here, $x_{t}$ takes the form

(73) $$x_{t}={\left[h_{0,0},\cdots h_{i,j},\cdots ,h_{n,m}\right]}^{T}.$$

The subscript *t* indicates terrain. The additional terrain state estimates are appended to the state vector as follows:

(74) $$x=\left[\begin{array}{c} x_{p}\\ x_{t} \end{array}\right].$$

As the terrain model is now part of the state vector, the LiDAR observation function is of the form

(75) $$z_{L}=h_{L}\left(x\right)+v_{z,L}.$$

The measurement function used to estimate the terrain and pose is identical to that of the previous section. However, expected range measurements are now determined through ray-casting onto the triangulated height grid model constructed from the $x_{t}$ vector, not a provided static model. The *z*-value or height of the vertices of each triangle of the grid, $\alpha_{j}$, $\beta_{j}$ and $\gamma_{j}$, are now defined by the elements of $x_{t}$. The measurement Jacobian for the terrain states is determined analytically from the partial derivative of the LiDAR range measurement with respect to the *z*-value of the points of the intercepted triangle ($\alpha_{j}$, $\beta_{j}$ and $\gamma_{j}$).

**Figure 13:**
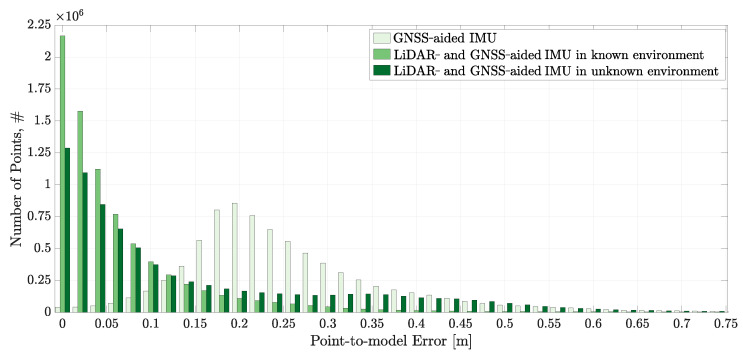
The point-to-model error distributions obtained by comparing the LiDAR endpoints with the ground truth terrain model from the three navigation solutions presented in this paper.

Figure 13 shows that the concurrent terrain and pose estimation system performs slightly worse than the LiDAR- and GNSS-aided IMU solution and significantly better than the GNSS-aided IMU solution. The mean point-to-model error for the concurrent terrain and pose estimation system is 0.14 m. The zero point-to-model error bin is the largest bin, with the error distribution extending to higher point-to-model error values.

**Figure 14:**
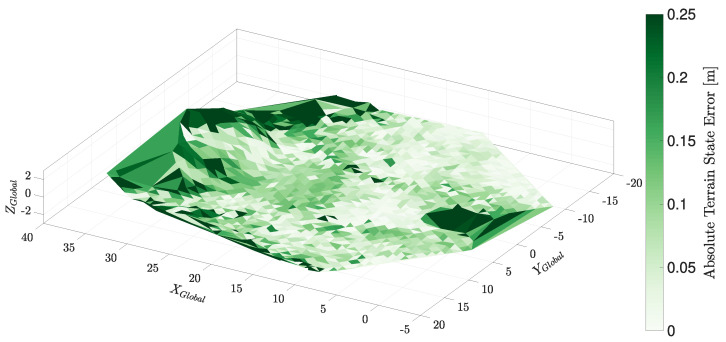
The error of the triangulated state mesh constructed whilst localising the platform. Here, the error of the terrain model is generally less than 0.05 m; however, there are some regions of larger error. The terrain estimates pictured here are fit for the purpose of terrain mapping in the use case this paper targets.

Figure 14 shows the environment model generated by the concurrent terrain and pose estimation system. The terrain model is coloured based on the difference between the estimated terrain and the surveyed ground truth model. The terrain estimate shows close agreement with the truth model: generally, state errors are less than 0.1 m but extend out to 0.25 m. The terrain estimate has an RMS error of 0.126 m, which was achieved using a 1 m height grid terrain representation. Generally, areas of large error are present in high gradient locations of the terrain model. These can be difficult to capture using a regular grid terrain representation.

**Figure 15:**
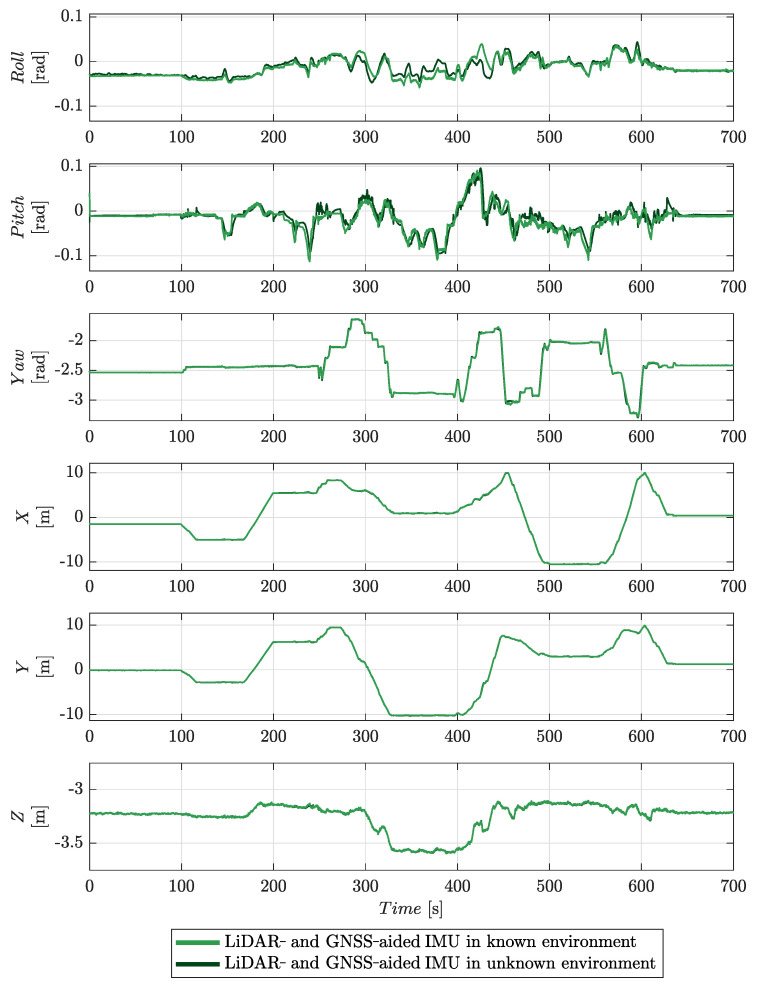
A comparison of the results from the LiDAR- and GNSS-aided IMU navigation solution both with and without an *a priori* environment model.

The estimates of the six degrees of freedom of the platform’s pose are shown in Figure 15. The solution generated without *a priori* knowledge of this environment shows some deviation in both roll and pitch when compared to that obtained using a known environment. The forward-looking orientation of the LiDAR sensor clearly stabilises the pitch estimate; the roll estimate is similar to the GNSS-aided IMU solution. A wider three-dimensional scan is expected to yield better stabilisation of roll. Equally, better roll estimates could be achieved by increasing the space between the GNSS antennas. The yaw, *x*, *y*, and *z* degrees of freedom are similar to those estimated when the environment is known. Overall, the results demonstrate the effectiveness of the approach.

The concurrent terrain and pose estimation filter, again, required more computing per timestep. It was demonstrated to run at ∼ 50 Hz on the target hardware (see Section 8).

## 10. Conclusions

The main result from this work is to show how LiDAR can be used to stabilise two-antenna GNSS-aided IMU pose estimators in environments with known and unknown geometry. The formulation is structured to accommodate multi-rate and non-synchronous measurements. It enables continuity through short-term periods of lost measurements from one of the sensors. Lost measurements are a characteristic observed on some brands of RTK-GNSS receivers, and the solution described in this paper adapts well to this situation.

We have not explored the possibility of using LiDAR stabilisation in GNSS-denied environments; however, the approach described here could be readily adapted to such situations.

In the paper introduction, we identified that LiDAR- and GNSS-aided IMU solutions can be effective when three-dimensional LiDAR measurements are available, but that three GNSS receivers are impractical or cost-prohibitive. Our recommendation, based on experience, is that if three receivers are possible, they should be used, particularly if an accurate pose solution to high frequencies is required. 

## Figures and Tables

**Table 1:** Specifications of the LiDAR, IMU, and GNSS sensors used as inputs to the navigation system of this paper.

Sensors					
**Velodyne VLP-16 LiDAR Sensor [41]**		**IMU**		**GNSS Receivers**	
		**Accelerometers**			
Number of rays:	16	Axes:	3	x uncertainty (1σ):	0.03 m
Range uncertainty (1σ):	0.03 m	Data Rate:	50 Hz	y uncertainty (1σ):	0.03 m
Horizontal field of view:	360${}^{\circ }$			z uncertainty (1σ):	0.055 m
Vertical field of view:	30${}^{\circ }$ (± 15${}^{\circ }$)			Signals tracked:	L1, L2, L5
Horizontal resolution:	0.1${}^{\circ }$– 0.4${}^{\circ }$	Gyroscopes		Data rate:	10 Hz
Vertical resolution:	2${}^{\circ }$	Axes:	3	Corrections:	RTK
Scan rate:	5–20 Hz	Data Rate:	50 Hz		
Points per second:	300,000				

**Table 2:** The diagonal elements of the IMU measurement covariance matrix. The cross-terms are several orders of magnitude smaller than the diagonal element. These values were determined experimentally from statistical analysis of a stationary data segment of 930 s.

IMU Covariance, $\sigma_{z,I}^{2}$					
${\ddot{x}}_{I}{\left[\frac{m}{s^{2}}\right]}^{2}$	${\ddot{y}}_{I}{\left[\frac{m}{s^{2}}\right]}^{2}$	${\ddot{z}}_{I}{\left[\frac{m}{s^{2}}\right]}^{2}$	${\dot{\theta }}_{I}{\left[\frac{{}^{\circ }}{s^{2}}\right]}^{2}$	${\dot{\phi }}_{I}{\left[\frac{{}^{\circ }}{s^{2}}\right]}^{2}$	${\dot{\psi }}_{I}{\left[\frac{{}^{\circ }}{s^{2}}\right]}^{2}$
$7.8\times 10^{-5}$	$3.9\times 10^{-5}$	$4.0\times 10^{-4}$	$7.6\times 10^{-3}$	$7.6\times 10^{-3}$	$1.4\times 10^{-2}$

**Table 3:** The diagonal elements of the GNSS measurement covariance matrix. The off-diagonal terms are several orders of magnitude smaller than the diagonal elements. These values were determined from statistical analysis of a 930 s measurement sequence where the platform was stationary.

GNSS Covariance, $\sigma_{z,G}^{2}$		
$x_{G}{\left[m\right]}^{2}$	$y_{G}{\left[m\right]}^{2}$	$z_{G}{\left[m\right]}^{2}$
$1.9\times 10^{-6}$	$2.4\times 10^{-6}$	$2.2\times 10^{-5}$

## Abbreviations

The following abbreviations are used in this manuscript:

DC	Direct current, referring to the zero-frequency component of a signal.
DOF	Degree(s)-of-freedom
EKF	Extended Kalman filter
EIF	Extended information filter
FIM	Fisher information matrix
FIV	Fisher information vector
GNSS	Global Navigation Satellite System
IEKF	Indirect extended Kalman filter
IMU	Inertial measurement unit
LiDAR	Light detection and ranging
RMS	Root mean squared
RTK	Real-time Kinematic
TTT	Track-type tractor
